# Therapeutic Notes

**Published:** 1904-12

**Authors:** Nelson W. Wilson

**Affiliations:** Buffalo, N. Y.


### PROGRESS IN MEDICAL SCIENCE.

### Therapeutic Notes.

Conducted by NELSON W. WILSON, M. D., Buffalo, N. Y.

### FOR COUGH IN CHILDREN.

At Roosevelt Hospital the following prescription is used with remarkably good results in the cough of children:

| | | | |
|---|---|---|---|
| ℞ | Ammonium chlorid | 8.00 | (ʒii.) |
| | Paregoric | 15.00 | (℥ss.) |
| | Syrup of squills | 15.00 | (℥ss.) |
| | Syrup of tolu, to make | 120.00 | (℥iv.) |

Mix. Dose: One teaspoonful every 2 or 3 hours.

### FOR LUMBAGO.

Solis-Cohen's internal treatment for lumbago is this prescription:

| | | | |
|---|---|---|---|
| ℞ | Salicylate of soda | 15.00 | [℥ss.] |
| | Iodide of potash | 8.00 | [ʒii.] |
| | Comp. syrup of sarsaparilla | 30.00 | [℥i.] |
| | Water, to make | 90.00 | [℥iii.] |

Mix. Dose: Teaspoonful in water 3 times a day, after meals.

### THE DIARRHEAS OF INFANCY.

Rotch, writing in *American Medicine*, believes the treatment of diarrheas of infancy is still largely empirical, but is of the opinion that it can be determined whether the large or small intestine is affected. The best success is obtained if the treatment is given by mouth, when the small intestine is the seat of disease, and by rectum, when the large intestine is affected. He differentiates the group of cases with marked fermentation and little local lesion from the ileocolitis cases in which the lesions are more or less decidedly marked. No specific remedy or intestinal antiseptic has been found to absolutely kill the organisms or neutralise their toxins. Clear the intestines of bacteria by laxatives, support the strength, combat nervous symptoms and hyperpyrexia are the chief indications. Of the drugs he recommends bismuth after the alimentary canal is free of all irritants:

| | | | |
|---|---|---|---|
| ℞ | Subnitrate of bismuth | 8.00 | [ʒii.] |
| | Salol | 1.00 | [gr. xvi.] |
| | Cinnamon water | 8.00 | [ʒii.] |
| | Water, to make | 30.00 | [℥i.] |

Mix. Dose: Teaspoonful in water every two hours. "Shake" label.

---

### HORSE-NETTLE FOR EPILEPSY.

Dr. I. Brower, in the *Texas Courier-Record of Medicine*, suggests the following combination in epilepsy, though large doses of bromide cause, as a rule, a very troublesome acne. To prevent this, a small dose of arsenic in the form of Fowler's solution may be added:

| | | | |
|---|---|---|---|
| ℞ | Bromide of soda | 40.00 | [ʒx.] |
| | Ext. solanum carolinense | 60.00 | [℥ii.] |
| | Camphor water, to make | 120.00 | [℥iv.] |

Mix. Dose: Teaspoonful 3 times a day in water.

---

### FOR A FAILING APPETITE.

The following is credited to Hemmeter as a spur to a flagging appetite:

| | | | |
|---|---|---|---|
| ℞ | Strychnine sulfate | .065 | [gr.i.] |
| | Dilute hydrochloric acid | 4.00 | [ʒi.] |
| | Elixir of gentian | 180.00 | [℥vi.] |

Mix. Dose: Half an ounce in half a glass of water through a tube.

It is a good prescription, but one which is better and which achieved remarkable results as an appetite-whip and a general tonic, is this which was thoroughly tested during the convalescent period following the Spanish war:

| | | | |
|---|---|---|---|
| ℞ | Tr. nux vomica | 20.00 | [ʒv.] |
| | Comp. tr. gentian | 50.00 | [ʒxii ss.] |
| | Comp. tr. cinchona | 50.00 | [ʒxii ss.] |

Mix. Dose: Teaspoonful in a little water half an hour before each meal.

The mixture is bitter, but not unpleasantly so. It will not be improved in taste by the addition of fluid extract of cascara, but in those cases where the latter drug is indicated it improves the prescription wonderfully.

